# Serum visinin-like protein-1 levels in preeclampsia: a cross-sectional pilot study

**DOI:** 10.1186/s12884-026-09181-3

**Published:** 2026-04-30

**Authors:** Neslihan Aslan, Cigdem Akcabay, Necip Ilhan, Miray Onat, Ebru Celik Kavak, Ibrahim Batmaz, Salih Burcin Kavak

**Affiliations:** 1 Gynecology and Obstetrics Clinic, Fethi Sekin City Hospital, Elazig, Turkey; 2 Department of Obstetrics and Gynecology, School of Medicine, Firat University Faculty of Medicine, Elazig, 23000 Turkey; 3 Department of Medical Biochemistry, Firat University Faculty of Medicine, Elazig, Turkey; 4 Gynecology and Obstetrics Specialist, Elazig, Turkey; 5 Department of Obstetrics and Gynecology, Faculty of Medicine, Mardin Artuklu University, Mardin, Turkey

**Keywords:** Preeclampsia, Visinin-like protein-1, Disease severity, Biomarkers, Pregnancy

## Abstract

**Background:**

To evaluate serum Visinin-like protein-1 (VILIP-1) levels in normotensive pregnant women and in patients with preeclampsia across the severity spectrum, and to investigate the association between VILIP-1 levels and disease severity.

**Methods:**

In this prospective cross-sectional pilot study, a total of 60 pregnant women were allocated into three groups: normotensive controls (*n* = 20), preeclampsia without severe features (*n* = 20), and preeclampsia with severe features (*n* = 20). Baseline clinical and laboratory characteristics were recorded. Serum VILIP-1 levels were measured and compared among groups. Statistical analyses were performed using non-parametric tests with adjustment for multiple comparisons.

**Results:**

Baseline demographic and obstetric characteristics were comparable among groups. Gestational age at delivery and neonatal birth weight were significantly lower in the preeclampsia with severe features group. Markers of hepatic and renal dysfunction were significantly elevated in this group, whereas hematological parameters did not differ significantly. Serum VILIP-1 levels were significantly higher in patients with preeclampsia with severe features compared with both normotensive pregnant women and those with preeclampsia without severe features (24.75 ± 18.27 ng/mL vs. 10.23 ± 7.98 ng/mL and 7.91 ± 4.97 ng/mL, respectively; adjusted *p* < 0.05).

**Conclusions:**

Serum VILIP-1 levels are significantly increased in preeclampsia with severe features and appear to be associated with disease severity and maternal organ involvement. These findings suggest that VILIP-1 may reflect disease severity rather than serving as a diagnostic biomarker; however, larger prospective studies are warranted to clarify its clinical utility.

## Background

Preeclampsia (PE) is a complex, pregnancy-specific hypertensive disorder affecting approximately 2–8% of pregnancies and remains one of the leading causes of maternal and perinatal morbidity and mortality worldwide [1]. The pathophysiology of preeclampsia is characterized as a multistep and progressive process initiated by abnormal placentation and impaired uteroplacental perfusion, followed by systemic endothelial dysfunction, oxidative stress, and a pronounced inflammatory response in the maternal circulation [2]. Increasing evidence suggests that this systemic inflammatory and endothelial disturbance may extend beyond the peripheral vasculature and exert significant effects on the maternal central nervous system.

Endothelial dysfunction and inflammation in preeclampsia have been proposed to compromise blood–brain barrier integrity and alter cerebral vascular function. Indeed, Escudero et al. demonstrated impaired cerebral vascular function in mothers exposed to preeclampsia and in their offspring, while Friis et al. reported disrupted blood–brain barrier integrity together with alterations in central nervous system biomarkers in patients with preeclampsia [3, 4]. Despite these findings, clinically applicable circulating biomarkers that reliably reflect neuronal injury in preeclampsia remain lacking.

Visinin-Like Protein 1 (VILIP-1), a member of the neuronal calcium sensor protein family, plays critical roles in calcium-mediated intracellular signaling, regulation of neuronal activity, cellular growth and differentiation, and synaptic plasticity [5]. Predominantly expressed in the central nervous system, VILIP-1 has emerged as a potential biomarker of neuronal injury and neuroinflammation in various neurological conditions, including ischemic stroke, traumatic brain injury, and neurodegenerative disorders [6–9].

Although neurological complications of preeclampsia are well recognized, biomarkers capable of reliably reflecting central nervous system involvement and subclinical neuronal injury remain insufficiently defined. Given the prominent roles of inflammation, endothelial dysfunction, and potential blood–brain barrier disruption in preeclampsia, VILIP-1 may represent a biologically plausible candidate biomarker for its neurological component.

The aim of this study was to compare serum VILIP-1 levels between patients with preeclampsia and healthy normotensive pregnant women and to evaluate the potential contribution of this biomarker to the pathogenesis of preeclampsia as well as its association with disease severity. The primary objective of this study was to evaluate whether serum VILIP-1 levels are associated with disease severity and target organ involvement rather than to establish its role as a diagnostic biomarker.

## Materials and methods

### Study design and study population

This study was designed as a prospective, cross-sectional, observational, and comparative pilot study conducted between April 2022 and November 2022 at the Department of Obstetrics and Gynecology, Faculty of Medicine, Fırat University. The study was planned in accordance with the principles of the Declaration of Helsinki and was initiated following approval from the Fırat University Clinical Research Ethics Committee (*Approval No:* 2022/05–10). Written informed consent was obtained from all pregnant women included in the study. Although participants were prospectively enrolled and data were collected in a forward-looking manner, the study design was cross-sectional in nature, as all measurements, including serum VILIP-1 levels, were obtained at a single time point without longitudinal follow-up.

A total of 60 pregnant women were included in the study. The participants were divided into three groups: healthy normotensive pregnant women (Group 1, *n* = 20), pregnant women diagnosed with preeclampsia (Group 2, *n* = 20), and pregnant women diagnosed with preeclampsia with severe features (Group 3, *n* = 20). The diagnoses of preeclampsia and preeclampsia with severe features were established in accordance with the 2020 updated guideline of the American College of Obstetricians and Gynecologists (ACOG) [10].

According to these criteria, preeclampsia was diagnosed in women with previously normal blood pressure who, after 20 weeks of gestation, developed a systolic blood pressure ≥ 140 mmHg and/or a diastolic blood pressure ≥ 90 mmHg accompanied by proteinuria (≥ 0.3 g protein in a 24-h urine collection or 2 (+) protein on spot urine testing). In cases of preeclampsia with further elevation of blood pressure and/or proteinuria accompanied by maternal organ dysfunction, the condition was classified as preeclampsia with severe features. Preeclampsia with severe features was diagnosed in the presence of any of the following findings:

*Severe elevation in blood pressure:* Systolic blood pressure ≥ 160 mmHg and/or diastolic blood pressure ≥ 110 mmHg.

*Central nervous system manifestations:* New-onset headache unresponsive to acetaminophen and not attributable to alternative causes, visual disturbances, or other central nervous system abnormalities.

*Hepatic dysfunction:* Elevated ALT or AST levels (serum transaminases exceeding twice the upper limit of normal).

*Severe persistent right upper quadrant or epigastric pain:* Not explained by alternative diagnoses.

*Renal dysfunction:* Serum creatinine > 1.1 mg/dL or a doubling of serum creatinine concentration in the absence of other underlying renal disease.

Hematological abnormalities: Progressive thrombocytopenia with a platelet count < 100 × 10⁹/L.

### Pulmonary edema

#### Inclusion and exclusion criteria

Women aged between 18 and 45 years with a singleton pregnancy at or beyond 20 weeks of gestation were included in the study. Participants with a prior history of chronic hypertension, chronic kidney disease, diabetes mellitus, autoimmune disease, thyroid dysfunction, infection, or malignancy were excluded. In addition, women with multiple pregnancies, known fetal congenital anomalies, smoking or alcohol use, and those receiving anti-inflammatory or antioxidant medications were excluded from the study. These criteria were applied to minimize potential confounding effects and to ensure a more homogeneous study population.

### Clinical and demographic assessment

For all participants, demographic and clinical characteristics—including age, gravida, parity, gestational age, body mass index (BMI), systolic and diastolic blood pressure values, obstetric history, and current clinical findings—were recorded. Blood pressure measurements were obtained after at least 10 min of rest, using an appropriately sized cuff, in the seated position, and were recorded based on two measurements taken at least 4 h apart.

### Blood sample collection and storage

Venous blood samples were obtained from all participants via the antecubital vein in the morning after a minimum of 8 h of fasting. The collected blood samples were centrifuged at 3000 rpm for 10 min to separate the serum. Serum samples were stored at − 80 °C until the time of analysis. Prior to analysis, the samples were thawed at room temperature and were not subjected to repeated freeze–thaw cycles.

### Measurement of visinin-like protein-1 (VILIP-1)

Serum visinin-like protein-1 levels were measured using a commercially available enzyme-linked immunosorbent assay (ELISA) kit (Bioabb, Human Visinin Enzyme-linked Immunoassay Kit, Bioabb, China, Wuhan). All measurements were performed in accordance with the manufacturer’s instructions. Samples were analyzed in duplicate under blinded conditions. The intra-assay and inter-assay coefficients of variation were within acceptable limits. Results were expressed in ng/mL.

### Routine laboratory parameters

In all patients, complete blood count, liver function tests (AST, ALT), renal function tests (urea, creatinine), lactate dehydrogenase (LDH), urinary protein levels, and other routine biochemical parameters were analyzed in the hospital’s central laboratory using standard methods.

### Statistical analysis

Statistical analyses were performed using SPSS software (version 22.0; IBM Corp., Armonk, NY, USA). Continuous variables were expressed as mean ± standard deviation (mean ± SD). The distribution of data was assessed using the Kolmogorov–Smirnov test, and homogeneity of variances was evaluated with Levene’s test. Although continuous variables were expressed as mean ± standard deviation for ease of interpretation and consistency with the literature, non-parametric tests were preferred due to the non-normal distribution of several variables.

Differences among groups were analyzed using the Kruskal–Wallis analysis of variance. For variables demonstrating a statistically significant difference in the Kruskal–Wallis test, pairwise comparisons were conducted using the Mann–Whitney U test. To reduce the probability of type I error in multiple comparisons, Bonferroni correction was applied, and the adjusted level of statistical significance was set at *p* < 0.017. All statistical tests were two-tailed, and no missing data were present in the analyses.

### Power analysis

A retrospective (post hoc) power analysis was conducted for the primary endpoint of the study, namely serum VILIP-1 levels. Based on the observed group means and standard deviations, the effect size was calculated as Cohen’s f and was determined to be 0.63 (large effect). With a total sample size of 60 participants (*n* = 20 per group) and under the assumption of three groups, the statistical power of the study was calculated as 0.99 at a significance level of α = 0.05.

When Bonferroni correction was applied for multiple comparisons and α was set at 0.017, the statistical power was determined to be 0.98. The power analysis was performed based on the observed effect size to demonstrate the adequacy of the study in detecting differences among the groups.

Due to the pilot nature of the study, no a priori sample size calculation was performed, and the sample size was determined based on feasibility and the availability of eligible participants during the study period.

## Results

A total of 60 participants were included in the study (Group 1: *n* = 20, Group 2: *n* = 20, Group 3: *n* = 20). The groups were comparable in terms of age, gravida, parity, and body mass index (BMI), and no statistically significant differences were observed for these variables (all *p* > 0.05).

However, gestational age at delivery and neonatal birth weight were significantly lower in Group 3 (Preeclampsia with severe features) compared with Group 1 and Group 2 (*p* < 0.017 after Bonferroni correction). The demographic and obstetric characteristics of the groups are presented in Table 1.

**Table 1:** Comparison of demographic and obstetric characteristics among Group 1, Group 2, and Group 3

Parameters	Group 1 (*n* = 20)	Group 2 (*n* = 20)	Group 3 (*n* = 20)	*p* value
Age (years)	27.25 ± 5.84	31.20 ± 7.75	30.75 ± 7.45	NS
Gestational Age (weeks)	37.6 ± 1.81	36.20 ± 2.5	32.70 ± 3.6	*
Gravida (number)	2.00 ± 1.12	2.30 ± 1.26	1.85 ± 1.08	NS
Parity (number)	0.80 ± 1.00	1.15 ± 1.18	0.65 ± 0.87	NS
BMI (kg/m^2^)	26.40 ± 2.3	25.20 ± 1.9	25.60 ± 1.81	NS
Neonatal Birth Weight (grams)	3093.50 ± 395.61	2557.45 ± 795.11	1667.85 ± 813.22	*

Values are presented as mean ± standard deviation (SD)

*BMI* Body Mass Index, *NB* Newborn, *NS* Not Significant

*p* > 0.05, Kruskal–Wallis test; * *p* < 0.017, Mann–Whitney U test (after Bonferroni correction)

When complete blood count parameters were evaluated, no statistically significant differences were observed among the groups in terms of white blood cell (WBC), hemoglobin (Hb), hematocrit (Hct), and platelet (Plt) values (*p* > 0.05).

In contrast, analysis of biochemical parameters revealed significant intergroup differences in markers reflecting hepatic and renal function. Aspartate aminotransferase (AST), alanine aminotransferase (ALT), lactate dehydrogenase (LDH), blood urea nitrogen (BUN), and creatinine levels were significantly higher in Group 3 compared with Group 1 and Group 2 (*p* < 0.017 after Bonferroni correction). The wide distribution observed in AST and ALT values in Group 3 was consistent with the clinical heterogeneity within this group. The complete blood count and biochemical parameters of the participants are summarized in Table 2.

**Table 2:** Comparison of complete blood count and biochemical characteristics among Group 1, Group 2, and Group 3

Parameters	Group 1 (*n* = 20)	Group 2 (*n* = 20)	Group 3 (*n* = 20)	*p* value
WBC (× 10^3^)	10.43 ± 2.98	12.91 ± 4.65	13.04 ± 5.07	NS
Hb (g/dl)	11.58 ± 1.10	10.93 ± 1.50	11.06 ± 1.22	NS
Hct (%)	35.47 ± 2.91	33.61 ± 3.65	34.00 ± 3.90	NS
Plt (× 10^3^)	237.75 ± 59.01	278.70 ± 128.43	205.80 ± 87.48	NS
AST (U/L)	22.35 ± 6.99	23.15 ± 7.07	74.40 ± 99.53	*
ALT (U/L)	13.80 ± 6.33	18.10 ± 16.92	65.10 ± 99.74	*
LDH (U/L)	262.80 ± 66.70	295.10 ± 75.44	349.15 ± 110.30	*
Urea (mg/dL)	16.85 ± 4.35	22.81 ± 7.27	31.36 ± 15.98	*
Creatinine (mg/dL)	0.48 ± 0.07	0.56 ± 0.10	0.72 ± 0.45	*

Values are presented as *mean* ± *standard deviation (SD)*

*n* number, *NS* Not Significant, *WBC* White blood cell, *Hb* hemoglobin, *Hct* Hematocrit, *Plt* platelet, *AST* Aspartate aminotransferase, *ALT* Alanine aminotransferase, *LDH* Lactate dehydrogenase, *BUN* Blood urea nitrogen

*p* > 0.05, Kruskal–Wallis test, *p < 0.017, Mann–Whitney U test with Bonferroni correction

With respect to the primary endpoint of the study—serum Visinin-like protein-1 (VILIP-1) levels—a statistically significant difference was observed among the groups. Serum VILIP-1 concentrations in Group 3 were significantly higher compared with those in Group 1 and Group 2 (*p* < 0.017). In addition, VILIP-1 values in the preeclampsia with severe features group demonstrated a wider distribution. Serum VILIP-1 levels across the groups are presented in Table 3.

**Table 3:** Comparison of serum Visinin-Like Protein-1 levels among Group 1, Group 2, and Group 3

Parameter	Group 1 (*n* = 20)	Group 2 (*n* = 20)	Group 3 (*n* = 20)	*p* value
Visinin-Like Protein 1 (ng/ml)	10.23 ± 7.98	7.91 ± 4.97	24.75 ± 18.27^1^	*

Values are presented as *mean* ± *standard deviation (SD)*

*n* number, Intergroup comparisons were performed using the Kruskal–Wallis test

*p < 0.017, ^1^Mann–Whitney U test (after Bonferroni correction)

**Fig. 1:**
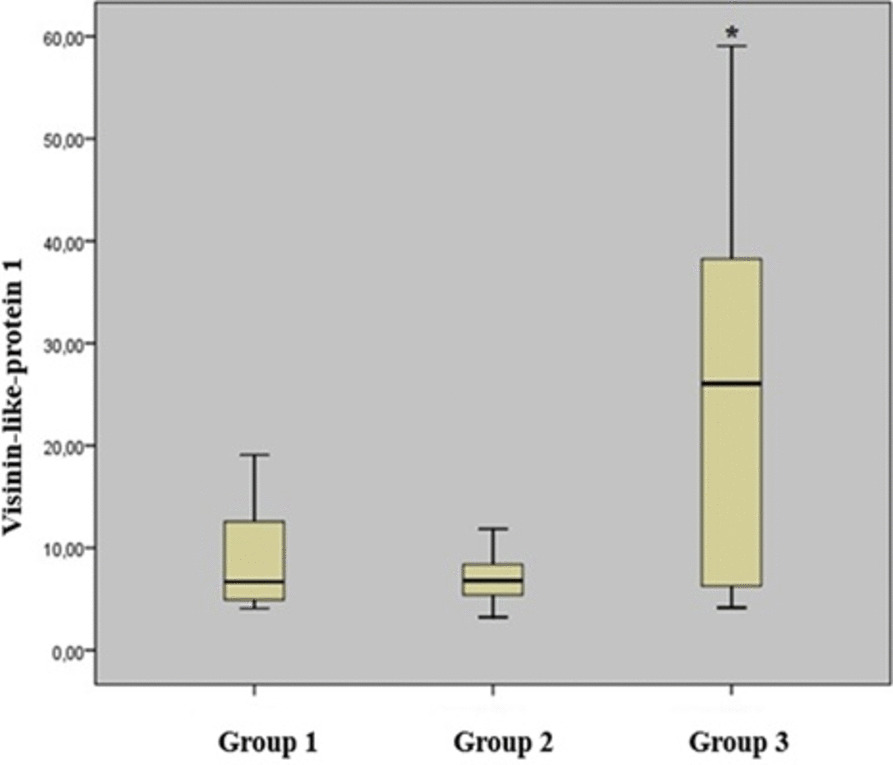
Distribution of visinin-like protein-1 levels across the study groups *. Indicates a statistically significant difference between median values, *p* < 0.017, Mann–Whitney U test

The distribution of VILIP-1 levels was visualized using a box-and-whisker plot. Median serum VILIP-1 values were higher and the distribution was wider in Group 3. The intergroup distribution and differences are illustrated in Fig. 1 (p < 0.017).

## Discussion

In this study, serum VILIP-1 levels were found to be significantly higher in patients with preeclampsia with severe features compared to both normotensive pregnant women and those with preeclampsia without severe features. The presence of earlier delivery, lower neonatal birth weight, and marked hepatic and renal dysfunction in the severe group suggests that the elevation in VILIP-1 levels may parallel disease severity. In contrast, the absence of significant differences in hematological parameters between groups indicates that alterations in VILIP-1 may be more closely associated with target organ involvement rather than systemic inflammation. The lack of a significant difference in serum VILIP-1 levels in the non-severe preeclampsia group may be explained by the earlier stage of the disease and the relatively limited extent of organ damage. Gestational age differences between groups may represent a potential confounding factor; however, we could not identify clear evidence in the current literature supporting an independent effect of gestational age on serum VILIP-1 levels. Therefore, the observed elevation in VILIP-1 levels appears to be more closely related to disease severity and maternal organ involvement.

In the limited number of studies investigating cerebrospinal fluid (CSF) in preeclampsia and eclampsia, increased levels of biomarkers associated with neuroinflammation and blood–brain barrier dysfunction have been reported; however, the correlation between CSF and peripheral circulating biomarker levels has been shown to be inconsistent [11, 12]. These findings suggest that serum biomarkers may reflect secondary changes accompanying central nervous system involvement rather than directly mirroring neural pathology. Moreover, women with a history of preeclampsia have been shown to have an increased risk of developing neurological disorders later in life, including stroke, dementia, and migraine [13, 14].

In a study conducted in 2025 by Bucher et al., a total of 129 women were included and categorized into four groups: 11 with eclampsia, 17 with preeclampsia complicated by end-organ involvement, 88 with uncomplicated preeclampsia, and 13 normotensive pregnant controls. Elevated CSF levels of claudin-5 and MMP-9 in women with eclampsia suggested disruption of blood–brain barrier integrity. In the same study, increased concentrations of pro-inflammatory cytokines were detected in CSF; however, no significant correlation was observed between CSF and plasma cytokine levels. These findings indicate that central nervous system involvement in preeclampsia, particularly in eclampsia, may be related to more complex mechanisms rather than directly reflecting the peripheral inflammatory response [15].Given the limited correlation observed between CSF biomarkers and serum markers, serum VILIP-1 levels may reflect central nervous system involvement indirectly rather than serving as a direct indicator of neural pathology.

VILIP-1 is a calcium-binding protein expressed in neuronal cells and plays a role in neuronal signal transduction and cellular stress responses. Elevated levels of VILIP-1 in blood and cerebrospinal fluid are considered reliable biomarkers of both acute and chronic neuronal injury (8,9).

In a recent study investigating VILIP-1 in pregnancy, cerebrospinal fluid (CSF) levels of VILIP-1, glial cell line–derived neurotrophic factor, and cadherin-6 were evaluated in women with pregnancy complications. VILIP-1 levels were found to be higher in women with complicated pregnancies compared to healthy pregnant controls, particularly among those with hypertensive disorders of pregnancy. Subgroup analysis demonstrated a 58% increase in VILIP-1 levels in hypertensive pregnancies, representing the most prominent biomarker alteration reported in the study. Notably, VILIP-1 was measured exclusively in CSF, and serum concentrations were not assessed. Furthermore, the study by Atalmis et al. was not limited solely to cases of preeclampsia [16]. In our study, serum VILIP-1 levels were evaluated in both subtypes of preeclampsia as well as in normotensive pregnant women. We found that serum VILIP-1 concentrations were associated with disease severity and were significantly higher in cases of preeclampsia with severe features. However, no statistically significant difference in serum VILIP-1 levels was observed between the overall preeclampsia group and the control group. In preeclampsia with severe features, widespread endothelial dysfunction and systemic inflammation may compromise the integrity of the blood–brain barrier, leading to increased permeability and neuroinflammatory processes. This disruption may result in subclinical neuronal injury and the subsequent release of neuronal proteins such as VILIP-1 into the systemic circulation. Therefore, elevated serum VILIP-1 levels may reflect central nervous system involvement and blood–brain barrier dysfunction associated with disease severity.

Although previous studies have not consistently demonstrated blood–brain barrier impairment or neuroinflammation in preeclampsia [17], emerging evidence suggests that central nervous system involvement may still occur and be reflected by circulating neuronal biomarkers. In this context, VILIP-1 appears to be a candidate marker of disease severity rather than a diagnostic marker of preeclampsia itself. Accordingly, VILIP-1 may be considered not as a tool to support the clinical diagnosis of preeclampsia, but rather as a potential biomarker for risk stratification and for guiding the intensity of clinical monitoring in affected patients. Recent evidence also suggests that thrombospondin-4 (TSP-4) may serve as a promising biomarker in preeclampsia, reflecting both disease presence and severity [18]. Elevated TSP-4 levels, particularly in early-onset and severe cases, have been associated with adverse perinatal outcomes, supporting its potential role in clinical risk stratification. These findings are consistent with our results, suggesting that VILIP-1 may similarly reflect disease severity rather than serving as a purely diagnostic marker.

The principal strength of this study lies in the comparative evaluation of serum VILIP-1 levels across three distinct clinical groups and the demonstration of a marked increase in the preeclampsia group with severe features. Although the specificity of elevated serum VILIP-1 levels for central nervous system injury could not be directly established, the findings may reflect the multisystem organ dysfunction observed in severe preeclampsia. Furthermore, the similarity of the groups with respect to baseline demographic and obstetric characteristics, together with adequate statistical power for the primary outcome, supports the reliability of the observed intergroup differences.

This study also has several limitations. Owing to its pilot design, the sample size was limited, which may have restricted the ability to detect smaller differences, particularly between the normotensive and non-severe preeclampsia groups. In addition, the single-center design and relatively small sample size may limit the generalizability of the findings to broader populations. In addition, VILIP-1 measurements were performed at a single time point, precluding the assessment of dynamic changes throughout pregnancy and the postpartum period. The absence of ultrasound and Doppler parameters may also be considered a limitation, as these assessments could provide additional information regarding disease severity. Detailed obstetric outcomes were not comprehensively evaluated, which may limit the interpretation of the relationship between VILIP-1 levels and perinatal outcomes. Furthermore, the cross-sectional design and limited sample size did not allow for multivariable regression modeling or receiver operating characteristic (ROC) analyses, which may preclude reliable assessment of the discriminative performance of VILIP-1. Therefore, the present findings should be interpreted as hypothesis-generating. Nevertheless, the findings suggest a potential role for VILIP-1 in preeclampsia with severe features and provide a foundation for larger, prospective, and multicenter studies.

## Conclusions

In conclusion, the findings of this pilot study demonstrate that serum VILIP-1 levels are significantly elevated in patients with preeclampsia with severe features compared to both normotensive pregnant women and those with preeclampsia without severe features. The association of increased serum VILIP-1 levels with earlier gestational age at delivery, lower neonatal birth weight, and marked hepatic and renal dysfunction suggests that this biomarker may be linked to disease severity. In contrast, the absence of significant differences in hematological parameters indicates that alterations in VILIP-1 may be more closely related to target organ involvement rather than systemic inflammation. Given the pilot and cross-sectional design of the study, predictive analyses were not intended; however, the present findings suggest that VILIP-1 may function less as a diagnostic marker of preeclampsia and more as a biomarker reflecting disease severity.

## Data Availability

The datasets used and/or analysed during the current study available from the corresponding author on reasonable request.
